# Combination of Albendazole and 2-Methoxyestradiol significantly improves the survival of HCT-116 tumor-bearing nude mice

**DOI:** 10.1186/1471-2407-13-86

**Published:** 2013-02-23

**Authors:** Anahid Ehteda, Peter Galettis, Krishna Pillai, David L Morris

**Affiliations:** 1 Cancer Research Laboratories, Department of Surgery, University of New South Wales, St. George Hospital, Sydney, NSW, Australia; 2 Cancer Pharmacology and Therapeutic Laboratory, Medical Oncology, St. George Hospital, Sydney, NSW, Australia

**Keywords:** Albendazole, 2-Methoxyestradiol, Combination therapy, Microtubule-targeting agents

## Abstract

**Background:**

Albendazole (ABZ) is a microtubule-targeting anthelmintic with a remarkable activity against a variety of human cancer cells. In this study, we examined if the antitumor activity of ABZ could be enhanced by its combination with other microtubule-binding agents.

**Methods:**

The interactions between ABZ and microtubule-binding agents, paclitaxel, vinblastine, colchicine, and 2-methoxyestradiol were characterized using median effect analysis method in HCT-116 colorectal cancer cells and DU145 prostate cancer cell line. The mechanism underlying the synergistic interaction related to tubulin polymerization and apoptosis was then investigated. Finally, the effect of the combination therapy on the survival of HCT-116 tumor-bearing nude mice was evaluated.

**Results:**

Among the tested drugs, a synergistic anti-proliferative effect was observed with the combination of low concentrations of ABZ plus colchicine and ABZ plus 2-methoxyestradiol (2ME). Exploring the mechanism of the interaction between ABZ and 2ME revealed that the combination therapy synergistically activated the extrinsic pathway of apoptosis. Consistent with *in vitro* results, the combination of low concentration of ABZ with 2ME prolonged the survival of mice-bearing HCT-116 tumors. High concentration of ABZ in combination with 2ME, however, proved to be less effective than ABZ alone.

**Conclusions:**

The combination of low doses of ABZ and 2ME has shown promising results in our pre-clinical model. Additionally, the finding that the combination of two microtubule-binding agents that share the same binding site can act synergistically may lead to the development of new therapeutic strategies in cancer treatment.

## Background

Combination therapy is the main approach in the treatment of various types of malignancies. The aims of using drug combinations are to increase efficacy, to reduce the dose and therefore, the toxicity, and to minimize or delay drug resistance [1].

Microtubule-targeting agents (MTAs) are among the most promising classes of the drugs in cancer therapy. Over the past decades, several MTAs have been discovered and developed into the established anticancer agents in the clinic. Traditionally, MTAs are classified into two major groups. One group are microtubule-stabilizing agents which polymerize microtubules and increase microtubule polymer mass. Second group, known as microtubule-destabilizing agents, inhibit polymerization of microtubules and reduce microtubule polymer mass [2]. Notwithstanding, at the clinically relevant concentrations, MTAs suppress microtubule dynamics, leading to the impairment of the metaphase to anaphase transition, and ultimately apoptotic cell death.

Given the fact that MTAs bind to different sites of tubulin, their combination with each other has potential to improve their antitumor activity. Additionally, combination therapy with MTAs may also reduce the toxicity associated with the use of one agent at its maximum tolerated dose (MTD). Currently, most chemotherapeutic drugs are administered as bolus injection at their MTDs followed by a rest period, allowing tumor cell re-population between successive chemotherapy [3]. Therefore, combining low doses of chemotherapeutic agents at close regular intervals may enhance efficacy while reducing the toxicity.

Albendazole (ABZ) is a microtubule-targeting agent from benzimidazole group of compounds. It has previously been shown that ABZ has a remarkable activity against a number of cancer cells [4,5]. In preclinical studies, ABZ suppressed the growth of solid tumors as well as the formation of malignant ascites derived from ovarian cancer [6]. Benzimidazoles have been shown to bind to the colchicine-binding site of tubulin and inhibit the polymerization of microtubules [7,8].

The aim of this study was to develop a novel therapeutic strategy using combination therapy. To this end, combination of ABZ with paclitaxel (PTX), vinblastine (VBL), and colchicine (CLC), as the representative drugs that interact with the established binding sites on tubulin was evaluated. Our findings showed that ABZ and CLC exhibited a synergistic anti-proliferative effect on HCT-116 and DU145 cell lines. Since CLC is not used in cancer therapy due to its toxicity [9], the interaction between ABZ and 2-methoxyestradiol (2ME), a related and a structurally similar compound to CLC was tested and found to be synergistic with ABZ. The mechanism underlying the synergistic interaction between ABZ and 2ME was then investigated and the antitumor effect of the combination therapy in mice bearing colorectal cancer xenograft was evaluated.

## Methods

### Chemicals

ABZ, 2ME, CLC, VBL, PTX, Sulforhodamine B, and carboxymethyl cellulose (CMC) were purchased from Sigma (Sydney, Australia), and hdroxypropyl-β-cyclodextrin (HPβCD) was from Cyclodextrin Technologies Development, Inc. (CTD; Florida, USA).

### Cell culture

The human colorectal cancer cell line HCT-116 and the prostate cancer cell line DU145 were originally obtained from ATCC. Both cell lines maintained in RPMI-1640 supplemented with 10% heat-inactivated fetal bovine serum (FBS), 50 units/ml penicillin, and 50 units/ml streptomycin. The cells were incubated at 37°C in a 5% CO_2_ humidified incubator.

### Cytotoxicity assay

HCT-116 and DU145 cells were seeded in 96-well plates at a density of 2500 and 3500 cells/well, respectively. After 24 hours incubation, the cells were treated with single agents and their combination for 72 hours. At the end of the treatment period, cell growth inhibition was assessed using the sulfothodamine B assay (SRB) as described previously [10].

To evaluate the effect of drug sequence in combination therapy, cells were treated with ABZ for 24 hours followed by 2ME for a further 48 hours or vice versa. In order to provide a constant experimental condition, concurrent treatment with ABZ and 2ME was performed alongside the sequential treatment and similarly, drug-containing medium was replaced with fresh drug after 24 hours.

### Drug interaction analysis

The interaction between the drugs in combination was determined by the median effect analysis using CalcuSyn software (Biosoft, Cambridge, UK), which calculates the combination index (CI) based on drug doses and cell survival. A CI less than 1 indicates synergism, equal to 1 indicates additivity, and greater than 1 indicates antagonism.

### Tubulin polymerization assay

HCT-116 cells were plated at a density of 5 × 10^5^ cells in six-well plates and allowed to attach overnight. Cells were then treated with ABZ, 2ME, and their combination. After 24 hours incubation with the drugs, a quantitative tubulin polymerization assay was performed as described previously [11].

### Western blotting

To prepare the whole cell lysate from cells, HCT-116 cells were grown to 60-70% confluency and treated with ABZ, 2ME or their combination for 24 hours. Cells were then lysed and centrifuged at 10,000 g for 10 minutes. To generate the lysate from tumor tissues, 100 mg tissue was lysed and homogenized in RIPA buffer (Sigma, Australia) containing 10% protease inhibitor cocktail. The samples were then centrifuged at 10,000g for 10 minutes and the protein content in supernatant was quantified using Bradford method (Bio-Rad protein assay kit, Bio-Rad, USA). Fifty micrograms protein were resolved on 12% gels and electrophoresed for 2 hours at 85V. Proteins were then transferred to Polyvinylidene Fluoride (PVDF) membranes and the membranes were incubated with the primary antibody for one hour at room temperature (Vascular Endothelial Growth Factor [VEGF] and P53, 1:200 dilution, Santa Cruz, USA) or overnight at 4°C (Death Receptor 5, [DR5] 1:400, R&D System, USA) followed by one-hour incubation with HRP-conjugated secondary antibodies (1:1000 dilution, Cell Signaling, USA). The bands were visualized by an enhanced chemiluminescence detection kit (GE Healthcare, Australia). The blots were then stripped using Seppro western blot stripping buffer (Sigma, Australia) and re-probed with β-actin (1:1000 dilution, Sigma, Australia).

### Caspase activity assay

To assess the activation of caspase 3, caspase 8, and caspase 9 in HCT-116 cells, the cells were incubated with ABZ, 2ME and their combination for 24 hours. The caspase activity was determined using caspase colorimetric assay kits according to the manufacturer’s instructions (R&D system, USA).

### Mice

Ten-week-old female BALB/c nude mice obtained from The Animal Resources Centre (Perth, Australia) and housed in a pathogen free environment for one week before the commencement of the experiments. All experiments were conducted according to the protocols approved by the Animal Experimentation Ethics Committee of the University of New South Wales.

### Drug preparation for in vivo study

ABZ was solubilized in 25% HPβCD (w/v in dH_2_O) [12]. 2ME was solubilized in 25% HPβCD (w/v in dH_2_O) containing 0.5% (w/v) carboxymethyl cellulose (CMC) at a concentration of 1 mg/ml. After 3 days storage at 4°C, 2ME was dissolved and no precipitation was observed.

### Tumor growth study

Prior to the *in vivo* experiment, a pilot study using two groups of four animals was carried out to evaluate the possible toxicity of simultaneous administration of ABZ and 2ME. Group 1 received the combination of ABZ and 2ME and group 2 were given the combination of the vehicle of the two agents. Following administration, mice in both groups showed an evidence of acute toxicity. Therefore, in the subsequent study, ABZ and 2ME were administered sequentially with ABZ preceding 2ME.

HCT-116 cells were harvested using 1% trypsin-EDTA and a single-cell suspension of 2×10^6^ cells in 0.1 ml of matrigel was injected subcutaneously into the hind leg of the animals. When the tumors had grown to approximately 100 mm^3^, the mice were randomized into six groups of 8–9 animals and treated intraperitoneally as follows: (1) 50 mg/kg ABZ, (2) 25 mg/kg ABZ, (3) 25 mg/kg 2ME, (4) 50 mg/kg ABZ + 25 mg/kg 2ME, (5) 25 mg/kg ABZ + 25 mg/kg 2ME, and (6) vehicle control. For combination treatment, ABZ was administered on day 1 followed by 2ME 24 h later (day 2). To assess the effect of individual drugs, the animals were treated with ABZ on day 1 and the vehicle of 2ME on day 2, or the vehicle of ABZ on day 1 and 2ME on day 2. Control animals received vehicle alone (ABZ vehicle on day 1 and 2ME vehicle on day 2). Tumor volume was measured every three days using the formula: (shortest diameter)^2^ x longest diameter × 0.5. When the tumor size reached 1000 mm^3^, mice were euthanized by an overdose of Lethabarb (Virbac, Australia).

### Immunohistochemistry

Immunohistochemistry analysis was performed as described previously [13]. For all experiments, multiple sections obtained from each treatment group and the average number of positive cells/area was calculated from 5 fields of each section.

Frozen sections (5 μm) were stained with CD31 (BD Bioscience, Australia) to visualize microvessels. For detection of apoptosis and proliferating tumor cells, paraffin-embedded sections (5 μm) were used. Apoptotic cells in tumor sections were stained using the terminal deoxynucleotidyl transferase–mediated dUTP nick end labeling (TUNEL) detection system (Roche) and proliferating tumor cells were detected by Ki67 staining (DAKO, Australia). Analysis of CD31-stained areas and TUNEL-positive cells was carried out using NIH ImageJ software (version 1.44; National Institutes of Health, Bethesda, MD) and Ki67-positive cells were quantified manually.

### VEGF ELISA assay

The concentration of VEGF in mice plasma was determined using Human VEGF Quantikine ELISA Kit (R&D System) according to the manufacturer’s instructions.

#### Statistical analysis

Data are presented as the mean ± SEM. All statistical analyses were performed using the GraphPad Prism software package version 5.0 (GraphPad Software Inc., San Diego, CA, USA). The survival days of animals were determined using the Kaplan-Meier plots and compared by the log-rank test. P values < 0.05 were considered to be significant. Differences between the groups were evaluated using Student’s *t-*test and one-way ANOVA.

## Results

### ABZ synergizes with colchicine

**Figure 1:**
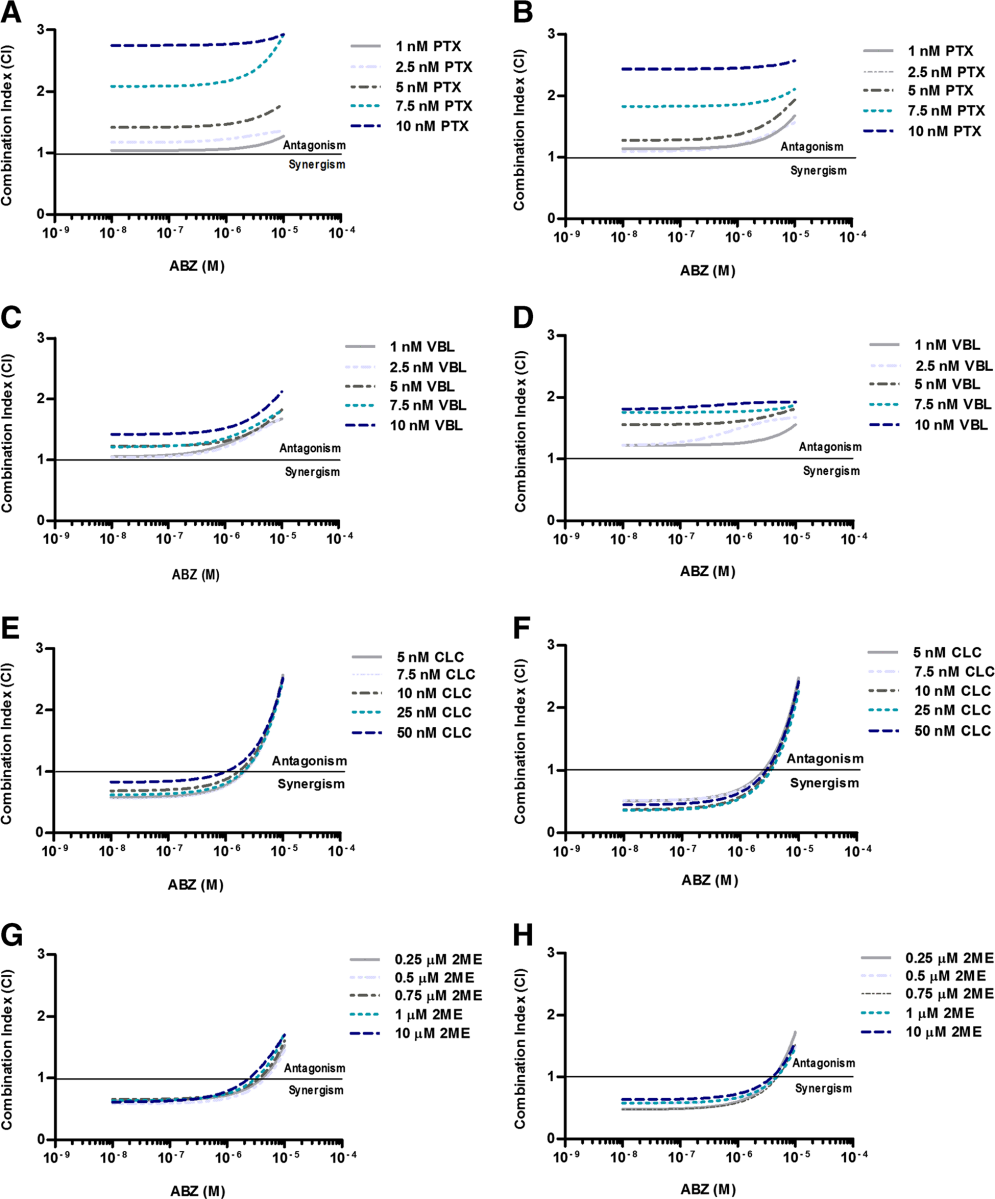
**The effect of ABZ in combination with PTX (A,B), VBL (C,D), CLC (E,F), and 2ME (G,H) on HCT-116 (left panel) and DU145 cells (right panel).** Cell viability was assessed by the SRB assay after 72 hours of simultaneous incubation with the drugs. Data were analyzed with Calcusyn software. CI < 1 indicates synergism, CI >1 indicates antagonism and CI = 1 shows additive effect.

In both HCT-116 and DU145 cells, the combination of ABZ and PTX were antagonistic, as CI values were consistently above 1 (Figure 1A and 1B). Likewise, antagonism was observed when ABZ combined with VBL, with CI values above 1 at all tested concentrations (Figure 1C and 1D).

Conversely, at lower concentrations (below 5 μM), ABZ was synergistic with CLC. However, as the concentration of ABZ increased, the interaction changed from nearly additive (5 μM ABZ) to antagonism (10 μM ABZ) (Figure 1E and 1F). Similar to CLC, the combination of ABZ and 2ME resulted in synergism at doses below 5 μM ABZ, as the combination index was consistently less than 1. However, the combination of 5 μM and 10 μM ABZ with 2ME led to additivity and antagonism, respectively (Figure 1G and 1H).

### Synergistic interaction between ABZ and 2ME is dose-dependent and schedule-dependent

**Figure 2:**
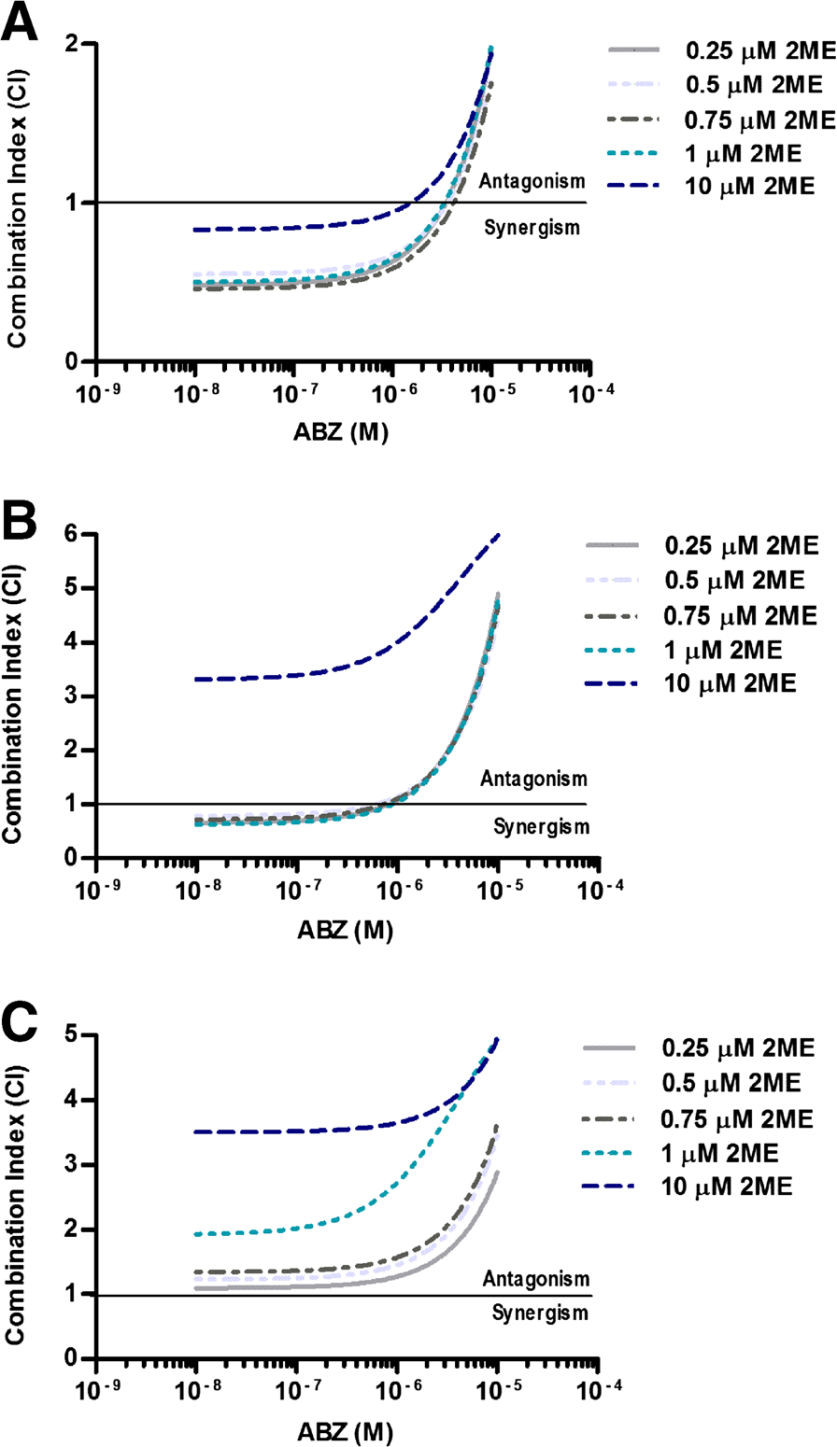
**Cytotoxic interaction between ABZ and 2ME. A**, HCT-116 cells were exposed to ABZ and 2ME for 72 hours simultaneously or **B**, treated with ABZ for 24 hours followed by additional 48 hours treatment with 2ME or **C**, treated with 2ME for 24 hours followed by additional 48 hours treatment with ABZ. CI < 1 indicates synergism, CI >1 indicates antagonism and CI = 1 shows additive effect.

We next evaluated the effect of drug sequencing on the synergism between ABZ and 2ME in HCT-116 cell line. As shown in Figure 2B, pre-treatment with ABZ resulted in higher CI values compared with simultaneous treatment (Figure 2A). Nevertheless, at concentrations below 5 μM ABZ, the interaction remained synergistic. As the concentration of ABZ increased, the interaction changed from synergism to antagonism. Pre-treatment with 2ME resulted in antagonism throughout the range of tested 2ME concentrations (Figure 2C). Indeed, as the concentration of 2ME increased, the antagonism was more pronounced.

### Mechanism of synergistic interaction between ABZ and 2ME

In the studies, the mechanism underlying the synergistic interaction between ABZ and 2ME was evaluated. To this end, the effect of the combination therapy on tubulin polymerization and apoptosis was investigated. The optimal concentrations of ABZ and 2ME in combination were determined in a 24-hours growth inhibition assay, and low concentrations, which had a minimal effect as single agents, were chosen.

### Combination of ABZ with 2ME has no synergistic effect on tubulin polymerization

**Figure 3:**
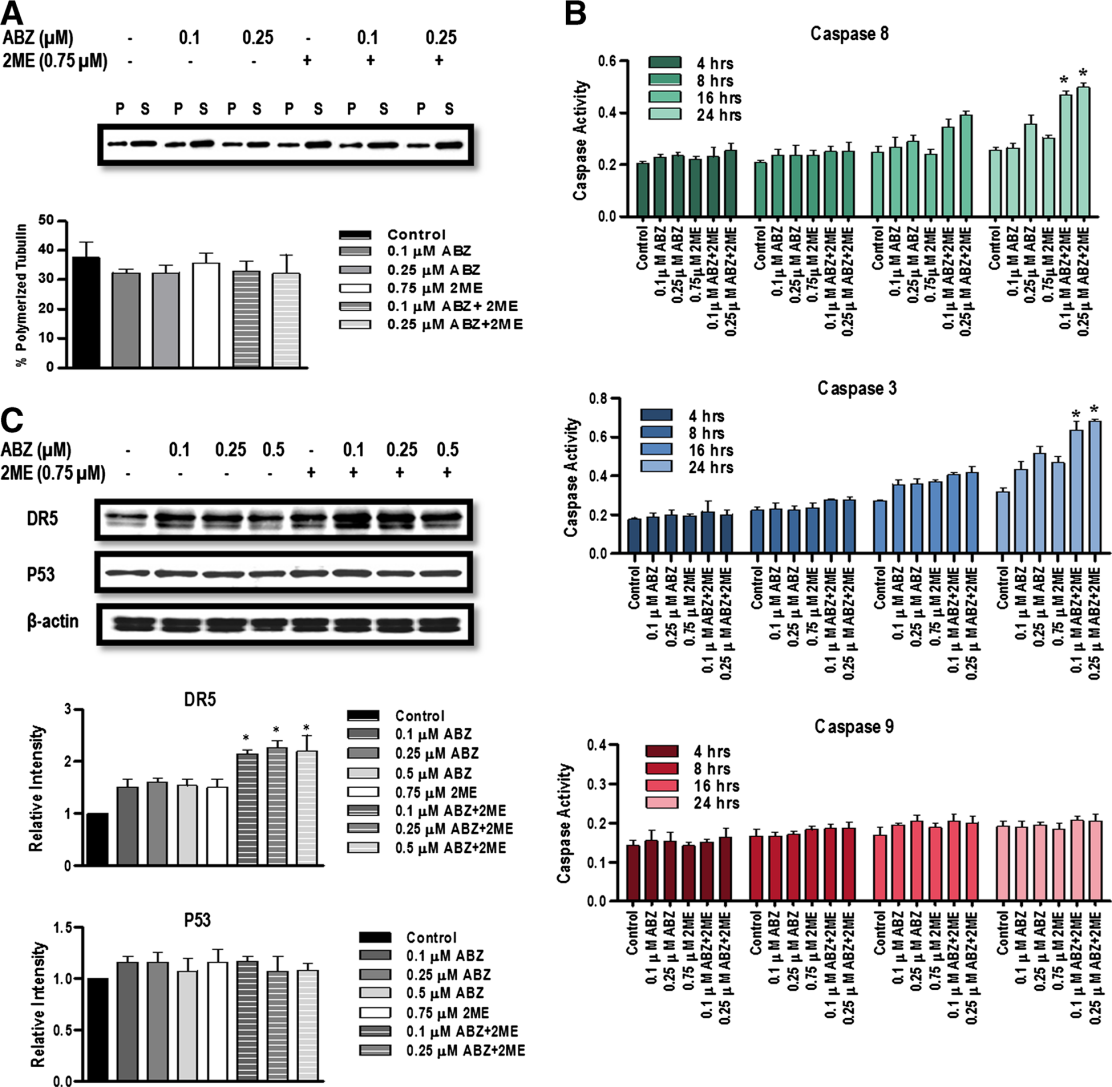
**Mechanism of interaction between ABZ and 2ME. A**, Effect of combination therapy on tubulin polymerization. Cells were treated with the indicated concentrations of ABZ, 2ME, and their combination for 24 hours The percentage of polymerized tubulin was determined by dividing the densitometric value of polymerized tubulin by the total tubulin content. The blot is a representative blot from four experiments. **B**, Effect of ABZ and 2ME on caspase activation. HCT-116 cells were incubated with vehicle, ABZ, 2ME, and their combination at indicated concentrations for 4, 8, 16, 24 hours. Caspase activity was determined by caspase substrates labeled with *p*-nitroaniline (*p*NA) using colorimetic assay. The data represent the mean values for duplicate measurements from three experiments. **C**, Effect of ABZ, 2ME and their combination on DR5 and P53 protein expression. HCT-116 cells were treated with vehicle, ABZ, 2ME, and ABZ plus 2ME at indicated concentrations for 24 hours. Cells were then lysed and subjected to western blot analysis. β-actin was used as a control for equal protein loading. The bar graphs represent the mean protein expression of three individual experiments, with error bars showing SEM. (*P < 0.05 compared with single agents).

To examine whether ABZ and 2ME synergistically depolymerize tubulin, a quantitative tubulin polymerization assay on HCT-116 cells was performed. In this assay, polymerized tubulin remains in the pellet after centrifugation, as it is not soluble in hypotonic buffer. In contrast, depolymerized or soluble tubulin remains in supernatant. Therefore, tubulin-depolymerizing agents increase the level of soluble tubulin in the supernatant whereas tubulin-polymerizing agents increase the insoluble tubulin in the pellet [14]. Treatment of HCT-116 cells with ABZ at the concentrations of 0.1 μM and 0.25 μM, and with 2ME at the concentration of 0.75 μM as single agents had no effect on tubulin polymerization compared with control (p > 0.05). Similarly, combination therapy did not increase tubulin depolymerization compared with the single agent treatment (Figure 3A).

### Combination of ABZ and 2ME induces apoptotic cell death in HCT-116 cells through extrinsic pathway

To investigate whether the combination of ABZ and 2ME activated caspase-dependent apoptotic pathway, caspase 8 and caspase 9 activities, as initiators of extrinsic and intrinsic pathway of apoptosis were evaluated. In addition, the activity of the downstream effector, caspase 3 was also assessed.

As depicted in Figure 3B, treatment with ABZ, 2ME and ABZ plus 2ME resulted in the activation of caspase-8 and caspase-3, which was evidenced after 16 hours. In cells that were treated with the combination of ABZ and 2ME, the activity of caspase-8 and caspase-3 was significantly higher in comparison with the single agents (p < 0.05). In contrast, neither the single agents nor their combination altered the activity of caspase-9, suggesting that the drug-induced apoptosis was not mediated through the intrinsic pathway (Figure 3B).

To further investigate the signaling events involved in the apoptosis, the effect of the combination treatment on DR5 protein levels was determined using western blot analysis. As shown in Figure 3C, ABZ and 2ME significantly upregulated the expression of DR5 compared with the vehicle-treated cells (p < 0.05). In addition, the combination of the two drugs further increased the levels of DR5 protein compared with the single agents (p < 0.05). These results suggest that the activation of caspase-8 is at least in part, dependent on death receptor signaling.

HCT-116 cells harbor wild-type p53. Therefore, it was hypothesized that the upregulation of DR5 could be a consequence of p53-induced growth arrest. To test this hypothesis, the level of p53 protein was determined by western blotting. As shown in Figure 3C, no significant difference in p53 levels was observed between control and treated cells, suggesting that the drug-induced cell kill, as well as upregulation of DR5 receptor was independent from p53.

### Combination of low doses of ABZ plus 2ME delays the tumor growth and prolongs the survival of mice-bearing HCT-116 xenograft

**Figure 4:**
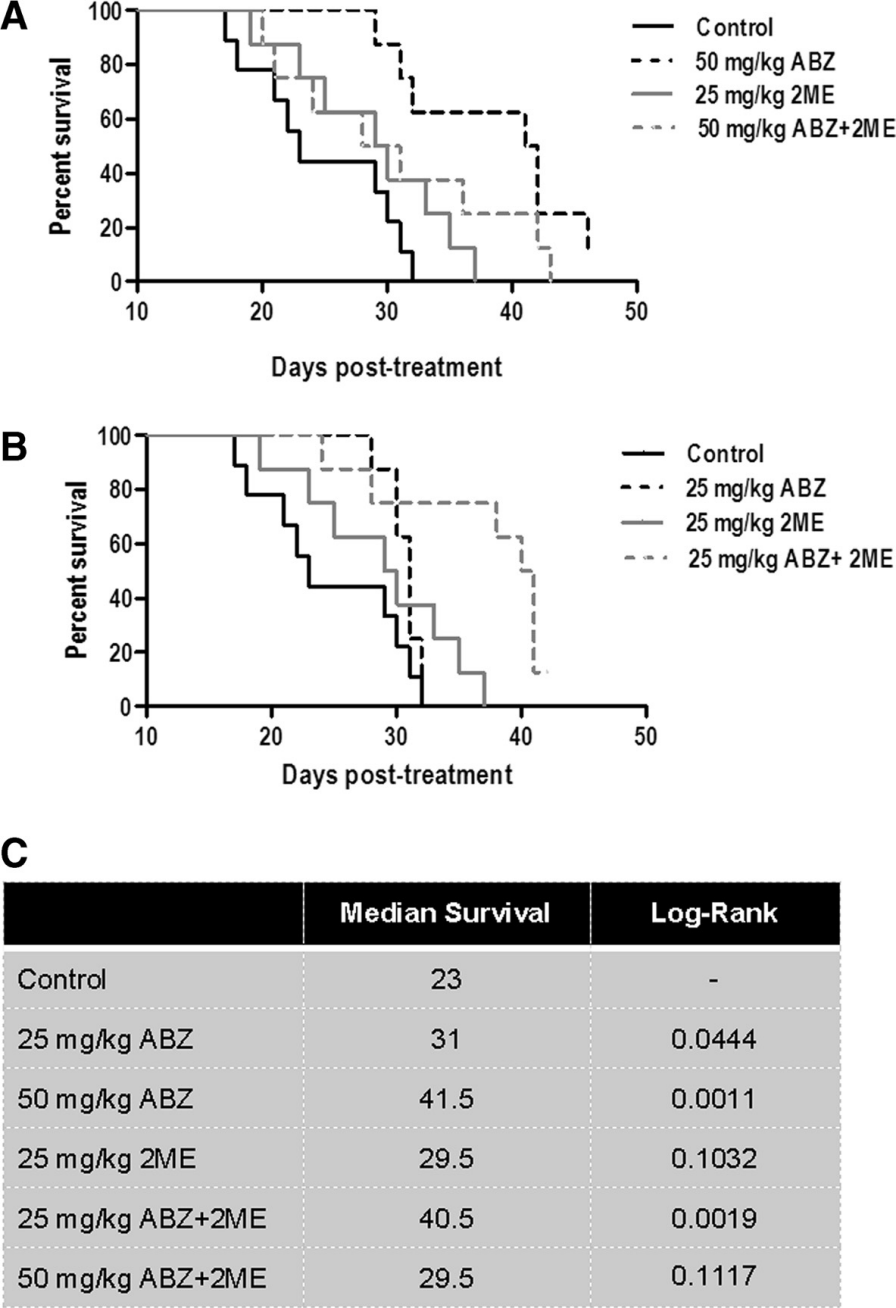
**In vivo response of HCT-116 xenografts to ABZ, 2ME and ABZ plus 2ME.** Mice were inoculated with 2x10^6^ HCT-116 cells. When the tumor size reached approximately 100 mm^3^, animals were randomized into six treatment groups and treated with the vehicle control, 25 mg/kg ABZ, 50 mg/kg ABZ, 25 mg/kg 2ME, 50 mg/kg ABZ plus 2ME, and 25 mg/kg ABZ plus 2ME. To assess the effect of individual drugs, animals were treated with ABZ on day 1 and the vehicle of 2ME on day 2, or ABZ vehicle on day 1 and 2ME on day 2. Combination therapy groups received ABZ on day 1 followed by 2ME 24 h later (day 2). Control animals received vehicle alone. Mice were euthanized when the tumor size reached 1000 mm^3^. (**A**) Effect of the combination of high concentration of ABZ (50 mg/kg) plus 2ME on the survival of the animals (**B**) Effect of the combination of low dose of ABZ (25 mg/kg) plus 2ME on the survival of the animals (**C**) Median survival analysis. The median survival of mice in all treatment groups was calculated using Kaplan-Meier statistics. The log-rank P-value is the comparison between each treatment group with untreated mice.

Treatment with 50 mg/kg ABZ as a single agent significantly prolonged the survival of mice compared with vehicle-treated group, with a median survival of 41.5 days for ABZ and 23 days for control mice (p = 0.001) (Figure 4). Likewise, 25 mg/kg ABZ as a single agent significantly delayed the tumor growth compared with the vehicle-treated group, with a median survival 31 days (p = 0.044). In contrast, 25 mg/kg 2ME alone led to a modest, but not statistically significant improvement in survival (median = 29.5), compared with control mice (p = 0.103).

Similarly, no survival benefit was observed in animals that were treated with the combination of 50 mg/kg ABZ and 25 mg/kg 2ME in comparison with vehicle-treated animals (median survival = 29.5 days, p = 0.111). In fact, combination therapy exhibited an antagonistic response, as the median survival of the animals which were treated with the combination of 50 mg/kg ABZ and 2ME was significantly less than the survival of mice that were treated with ABZ as a single agent (p = 0.096) (Figure 4A). These results were consistent with *in vitro* drug interaction analysis where the combination of 2ME with high concentrations of ABZ resulted in an antagonistic effect on the proliferation of HCT-116 and DU145 cells (Figure 2).

In contrast, the combination of 25 mg/kg ABZ with 2ME statistically significantly prolonged the survival of animals (median = 40.5 days) in comparison with control group (p = 0.0019). In addition, the combination of 25 mg/kg ABZ with 2ME was substantially superior to ABZ and 2ME alone (p = 0.015 and p = 0.005, respectively) (Figure 4B).

### Combination of ABZ and 2ME reduces the proliferation rate and microvessel density and induces apoptosis in HCT-116 tumor

**Figure 5:**
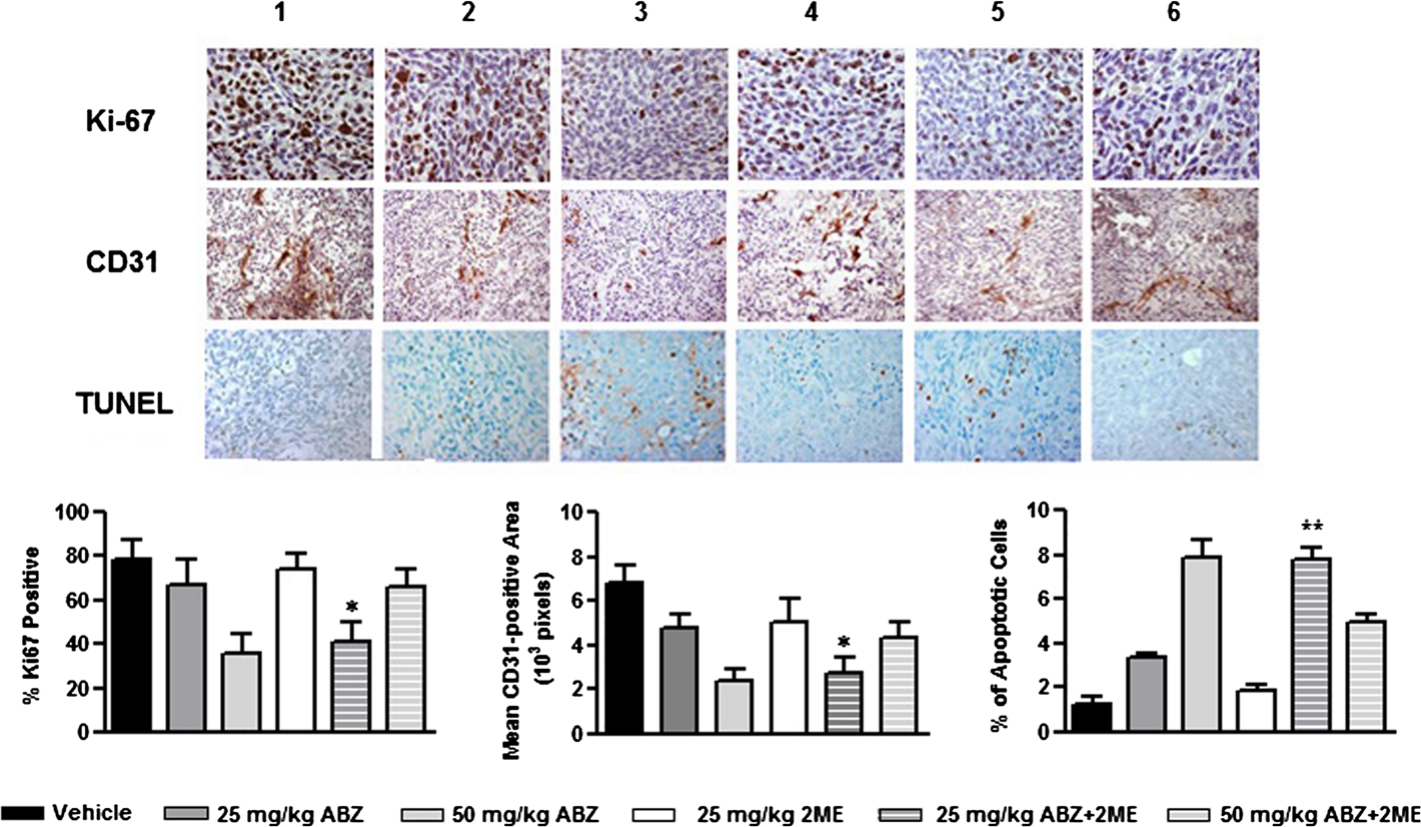
**Effect of ABZ, 2ME, and their combination on inhibition of tumor cell proliferation, angiogenesis, and apoptosis in HCT-116 subcutaneous tumor.** Ki67-positive cells (brown) were counted and reported as the percentage of the total number of cells in proliferative phases in each field. CD31-positive vessel area (brown) were measured per high-power field (10^3^ pixels). Cells underwent apoptosis (brown) were counted and reported as the percentage of total cells. (1) Vehicle control, (2) 25 mg/kg ABZ, (3) 50 mg/kg ABZ, (4) 25 mg/kg 2ME, (5) 25 mg/kg ABZ plus 2ME, and (6) 50 mg/kg ABZ plus 2ME. (*P < 0.05 and **P < 0.001 compared with single agents).

To assess the effect of the combination therapy on the suppression of tumor cell proliferation, immunohistochemistry analysis of Ki67 on tumor sections from all treatment groups was performed (Figure 5). While both 50 mg/kg and 25 mg/kg ABZ as single agents significantly reduced the proliferation rate of the tumor cells, 2ME had no effect on ki-67 rate. Combination therapy with 50 mg/kg ABZ and 2ME resulted in a significant decrease in the tumor proliferation rate in comparison with the control (p < 0.05). However, 50 mg/kg ABZ as a single agent was more effective in the suppression of the proliferation rate than the combination therapy (p < 0.05). Conversely, combination of 25 mg/kg ABZ with 2ME led to a statistically significant reduction in the proliferation rate, compared with vehicle-treated mice and the mice that received ABZ and 2ME as single agents (p < 0.05). These results suggest that the increase in survival rate of animals that were treated with the combination of 25 mg/kg ABZ and 2ME was, at least in part, due to the suppression of the tumor cells proliferation.

To examine whether the inhibition of tumor growth was associated with a reduction in the vascularization level of the tumors, the expression of CD31, an endothelial cell marker was evaluated using immunohistochemistry (Figure 5). Animals that were treated with single agents had a significant decrease in the tumoral CD31 compared with the mice that received no treatment (p < 0.05). Similarly, tumor sections from the mice that received the combination of 50 mg/kg ABZ with 2ME displayed a significant reduction in CD31 antigen in comparison with vehicle-treated group (p < 0.05). However, 50 mg/kg ABZ as a single agent was more effective in reducing CD31 levels compared with the combination therapy (p < 0.05). Additionally, no differences in the degree of vascularization were observed in combination-treated group in comparison with 2ME-treated group (P > 0.05). In contrast, in the animals that received the combination of 25 mg/kg ABZ and 2ME, suppression of angiogenesis was more pronounced than in mice that were treated with the single agents (p < 0.05). These results imply that the survival benefit of animals that were given the combination of 25 mg/kg ABZ and 2ME was in part, due to the suppression of angiogenesis.

Finally, TUNEL assay was performed to determine whether ABZ, 2ME and their combination induce apoptotic cell death in tumor cells (Figure 5). TUNEL-positive cells in tumors which were treated with ABZ alone at both 25 and 50 mg/kg concentrations were significantly higher than vehicle-treated tumors (p < 0.05). In contrast, the number of TUNEL-positive cells in the animals that were treated with 2ME was not significantly different from those that received no treatment (p > 0.05). Combination therapy with 50 mg/kg ABZ and 2ME was significantly less effective in inducing apoptosis in tumor cells, compared with the effect of 50 mg/kg ABZ as a single agent (p < 0.05). In contrast, in the animals that received the combination of 25 mg/kg ABZ and 2ME, TUNEL-positive cells were markedly higher than those that were treated with the single agents (p < 0.05).

These results suggest that in addition to the suppression of the proliferation rate and angiogenesis, induction of apoptosis was also contributed to the survival benefit of the animals that were treated with the combination of 25 mg/kg ABZ and 2ME.

### Combination of ABZ and 2ME suppresses VEGF in tumor and plasma of mice-bearing HCT-116 tumor

To further explore the mechanism underlying the interaction between ABZ and 2ME, tumors and plasma samples of the animals from each treatment group were analyzed for VEGF expression.

**Figure 6:**
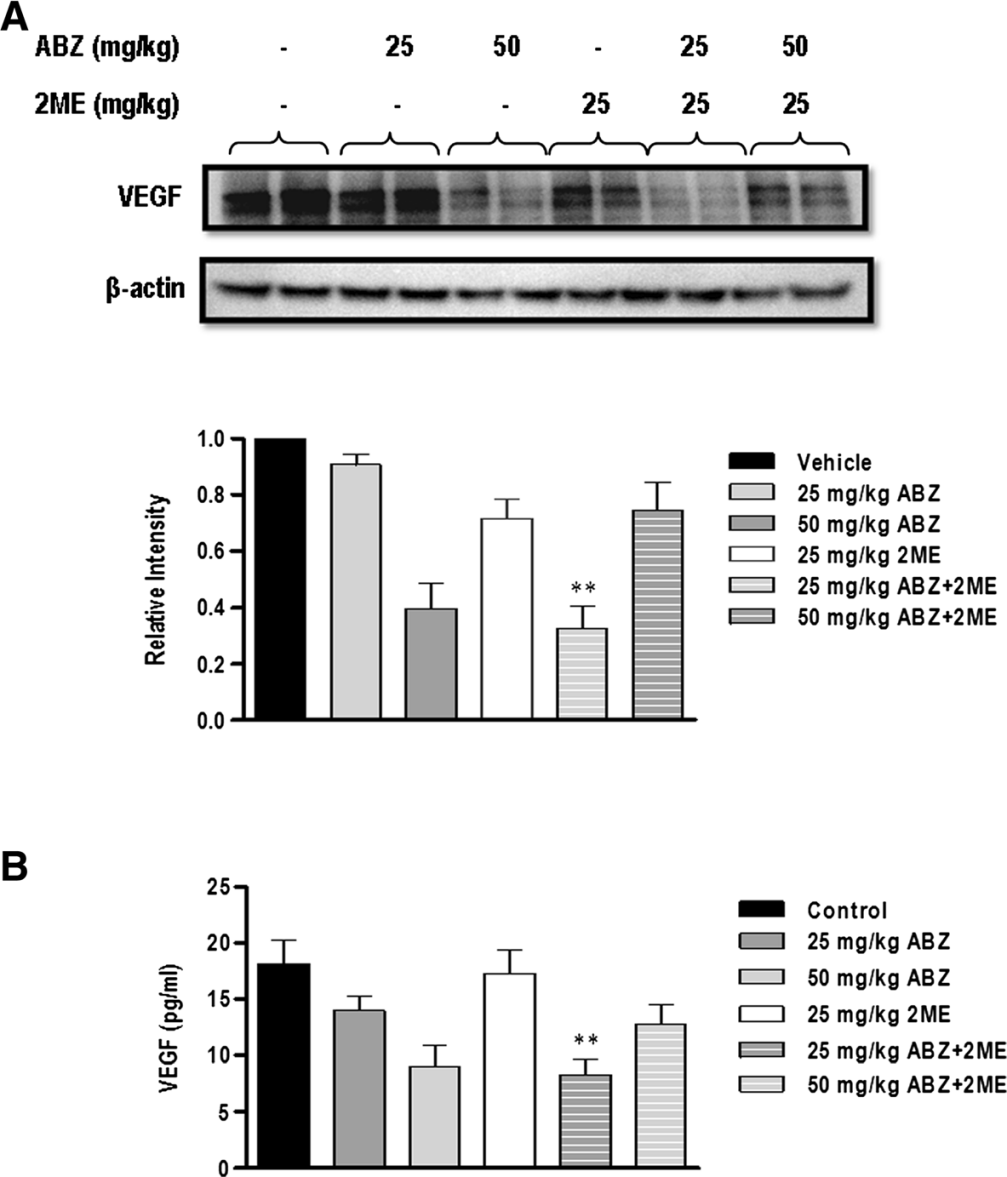
**Effect of ABZ, 2ME and combination on VEGF levels in tumor and plasma sample. A**, Four tumors from each treatment group were analyzed for VEGF levels and the blot is representative of four samples. β-actin was used as a control for equal protein loading. Graph shows densitometric analysis of VEGF immunoblot and each column represents the mean ± SEM. **B**, Following euthanasia, blood was collected by cardiac puncture and plasma samples were subjected to ELISA assay for VEGF levels. Each column represents mean VEGF levels ± SEM (n = 8–9). (**P < 0.001 compared with single agents).

As shown in Figure 6A, 50 mg/kg ABZ significantly reduced the expression of VEGF in tumor (p < 0.0001). In contrast, 25 mg/kg ABZ and 2ME as single agents had no significant effect on VEGF expression (p > 0.05). Consistent with the results from immunohistochemistry, the combination of 50 mg/kg ABZ and 2ME was less effective than 50 mg/kg ABZ as a single agent. Conversely, 25 mg/kg ABZ combined with 2ME markedly suppressed VEGF expression (p < 0.0001).

As depicted in Figure 6B, both 25 mg/kg ABZ and 2ME failed to reduce VEGF levels in plasma (p > 0.05). In contrast, in animals that were treated with 50 mg/kg ABZ, VEGF levels were significantly decreased (p = 0.0052). Combination therapy with 50 mg/kg ABZ and 2ME, had no effect on VEGF levels in comparison with vehicle (p > 0.05). However, a significant reduction in VEGF level was observed in the mice that received the combination of 25 mg/kg ABZ with 2ME compared with vehicle-treated group (p = 0.0016), and animals which were treated with ABZ and 2ME as single agents (p = 0.01 and p = 0.0025, respectively).

## Discussion

One of the main approaches in cancer therapy is to utilize the combination of chemotherapeutic agents with the objective of improving efficacy while maintaining the overall toxicity to an acceptable level. As a single agent, ABZ has been shown to be a promising anticancer agent both *in vitro* and *in vivo*. Nevertheless, its combination with other cytotoxic agents may further improve its application. Given the proven success of MTAs such as *vinca* alkaloids and taxanes in cancer therapy and the fact that MTAs can synergize with one another, the combination of ABZ with PTX, VBL, and CLC was evaluated.

In both HCT-116 and DU145 cell lines, the combination of ABZ with PTX resulted in antagonism throughout the PTX concentrations used. Likewise, VBL had an antagonistic interaction with ABZ regardless of the concentration tested. Surprisingly, a dose-dependent synergistic interaction between ABZ and CLC was observed. CLC is not being used in cancer treatment despite its effectiveness. Therefore, the interaction between ABZ with 2ME was evaluated. 2ME has been shown to be active against a variety of cancer cells both *in vitro* and *in vivo* and more importantly, it does not exhibit myelosupression and other hematological toxicities associated with MTAs [15]. This property makes 2ME an ideal candidate for the combination with other MTAs, as overlapping toxicities being the major limiting factor in combination therapies would be greatly diminished. It was hypothesized that since 2ME shares the same binding site on β-tubulin as CLC, and its structure and mechanism of action are similar to CLC, it may act synergistically with ABZ.

Similar to CLC, 2ME exhibited a dose- and schedule-dependent synergistic interaction with ABZ in inhibiting the proliferation of HCT-116 and DU145 cells. While simultaneous treatment with ABZ and 2ME resulted in the most synergistic interactions compared with other schedules, pre-incubation with 2ME led to antagonism in all tested concentrations.

Synergism between MTAs has been reported previously. For example, paclitaxel can act synergistically with vinblastine [11], and estramustine [16]. Estramustin plus vinblastine [17,18], and vinorelbine plus docetaxel [19] are more effective than either drug alone, and vinorelbine plus paclitaxel [20], and docetaxel plus CI-980 [21], are superior to single agents. Yet, it is uncommon for two agents to interact synergistically while they bind to the same binding site. More often, this kind of combination leads to additivity or antagonism, as the drugs cannot bind to the same site simultaneously. The only exception reported, is a study by Martello et. al., demonstrating that taxol and discodermolide represented a synergistic drug combination in four human cancer cell lines [22] and a subsequent study showed that taxol and discodermolide synergistically suppress the microtubule dynamics [23].

Several studies have reported that benzimidazoles bind to the colchicine-binding site of mammalian tubulin [7,8,24,25]. In contrast, a recent study suggested that benomyl, an antifungal agent and a member of benzimidazole compounds, did not inhibit the binding of colchicine to its binding site [26] and in a subsequent study, the combination of benomyl and colchicine was shown to be synergistic [27]. Due to these conflicting data, we investigated the interactions between 2ME plus CLC, and combretastatin A4 (CA4) plus CLC. In addition, the combination of these three colchicine-binding agents with one another was also evaluated (data not shown). It was hypothesized that antagonism between colchicine-binding agents would further confirm that ABZ does not bind to the colchicine-binding site. Surprisingly, we found that all four tested colchicine-domain binders represented a concentration-dependent synergistic cytotoxic effect on the proliferation of HCT-116 and DU-145 cell lines.

It is well known that at clinically relevant doses, both microtubule-polymerizing and microtubule-depolymerizing compounds suppress the dynamics of microtubule without affecting the microtubule polymer mass. For instance, the IC50 values for inhibiting the cell proliferation by 2ME is 10- to 100-fold lower than the concentration required for tubulin depolymerization [28]. As for ABZ, in 1A9 ovarian cancer cells the concentration required for tubulin depolymerization, is 10-fold higher than the IC50 values for inhibiting cell growth [29]. These results suggest that there is only a modest correlation between the concentrations that induce cell death and the concentrations that affect microtubule polymer mass. Similar to ABZ and 2ME, it has been shown that at low concentrations, CLC suppresses the dynamic instability of microtubules with no effect on the polymer mass [30]. As a result, while the stoichiometry of CLC binding to soluble tubulin is approximately 1 mol per mol tubulin [31] much less CLC with very little binding is required to inhibit the dynamic instability of microtubules [32]. Considering that each micrometer of microtubule contains 1690 tubulin dimers, a great number of binding sites are available for the drug molecules [9,33]. Therefore, it is conceivable that additional drugs could be bound to microtubules.

Another explanation is that the drugs may exhibit different affinity towards different isotypes of tubulin [34]. In mammals, six isotypes of α-tubulin and seven isotypes of β-tubulin have been recognized [35]. CLC has been proven to have the highest affinity for αβ_IV_ and the lowest affinity for αβ_III_[36]. Unfortunately, there is no published data on the binding affinities of ABZ and 2ME to various tubulin isotypes. Nevertheless, if the drugs discriminate among the various isotypes or among various forms of microtubules and the preference of each drug in terms of binding to a specific isotype of tubulin differs from the other drug, then it is plausible to conclude that the synergy may result from different affinity of the drugs towards different forms or isotypes of microtubules [34].

The mechanism of synergism between ABZ and 2ME was next explored by evaluating the effects of the combination therapy on tubulin polymerization and apoptosis-related proteins. As expected, comparison between the percentage of polymerized tubulin in the cells that were treated with the single agents and with the combination revealed that the concentrations at which synergistic anti-proliferative effect were observed, did not induce depolymerization of tubulin. These results imply that the effect of the combination of ABZ and 2ME on cell proliferation and their synergistic interaction is not mediated through the inhibition of tubulin polymerization. In this regard, it remains to be established whether the suppression of microtubule dynamics account for this synergistic interaction.

Activation of caspase cascade has been proven to be the central mechanism promoting apoptosis in response to chemotherapy. Therefore, to elucidate whether the cell death induced by ABZ and 2ME is mediated through apoptosis, the activity of caspase-3, caspase-8 and caspase-9 were assessed. Two major apoptosis pathways, the extrinsic pathway and the intrinsic pathway converge on caspases activation [37]. The extrinsic pathway is activated through the death receptors (DRs). Interaction of DRs with tumor necrosis factor-related apoptosis–inducing ligand (TRAIL) induces the activation caspase-8, which in turn, triggers the cascade of activated procapases that follows [37-39]. The intrinsic pathway is controlled by Bcl-2 family of proteins and involves the release of apoptosome containing caspase 9 [38]. Activation of initiator caspase-8 and −9 result in the cleavage of caspase-3 [40,41]. Results from caspase activity assay revealed that while both single agent treatment and combination therapy failed to activate caspase-9, they activated caspase-8 and caspase-3 in a time-dependent manner. Additionally, combination therapy was significantly more effective in the activating caspase-8 and caspase-3 in comparison with single drug treatment. The expression of DR5 was also induced in the cells that were treated with both single agents and the combination of the drugs, with combination treatment producing a more pronounced effect. These results suggest that the extrinsic death receptor apoptotic pathway is involved in drug-induced apoptosis in HCT-116 cells. The mechanism of upregulation of DR5 is not clear but most likely is independent form p53, as no increase in the protein levels of P53 was observed. DR5 has been shown to be activated in both ligand-dependent and ligand-independent manner. While p53 has been implicated in ligand-independent upregulation of DR5 [42], it is not required for the apoptotic response to TRAIL [43]. Possible role of TRAIL in DR5 overexpression and the upstream signal that lies between drug exposure and activation of caspases are unknown.

Historically, MTAs-induced apoptosis is mediated primarily by the mitochondria/caspase 9 activation pathway. Yet, it has been reported that the extrinsic pathway may also be engaged by MTAs. For instance, PTX has been shown to up-regulate DR5 protein and sensitizes prostate cancer cell lines to the cytotoxic effects of TRAIL [44]. However, these effects appear to be cell line-dependent, as they were not observed in non-small cell lung and breast cancer cell lines [45,46]. In agreement with current results, 2ME has also been reported to overexpress DR5 in a variety of human cancer cell lines including breast, cervical, prostate and glioma cells [47].

In our *in vivo* pilot study, concurrent administration of ABZ and 2ME resulted in an acute toxicity in the animals. Signs of toxicity were convulsion and haematuria which were also observed when the mice received the combination of the drug-free vehicles. This toxicity was at least in part, due to the high concentrations of HPβCD, as the LD50 of HPβCD in mice is 10 g/kg, while the combination of the vehicles contained nearly 12 g/kg HPβCD. Therefore, in the subsequent study, ABZ and 2ME were administered sequentially with ABZ preceding 2ME. Although this schedule showed no toxicity to the animals, it may have compromised the antitumor efficacy, as the results from in vitro drug interaction analysis suggested that simultaneous treatment with ABZ and 2ME was the most effective schedule.

Evaluating the combination therapy in mice-bearing HCT-116 tumors indicated that 50 mg/kg ABZ as a single agent was more effective in prolonging the survival of animals compared with combination therapy. These results were consistent with the *in vitro* assay where the combination of high concentrations of ABZ with 2ME represented an antagonistic anti-proliferative effect on HCT-116 cells. In contrast, combination of low dose of ABZ (25 mg/kg) with 2ME significantly prolonged the survival of mice compared with treatment with single agents. In addition, combination therapy significantly suppressed tumor cell proliferation compared with treatment with single agents. Further, analysis of tumor samples from these mice revealed that the combination therapy significantly decreased microvessel density and markedly increased the number of apoptotic tumor cells. Finally, tumor and plasma of mice that were treated with the combination of ABZ and 2ME had a significantly lower VEGF levels in comparison with the animals that received single agents or vehicle.

## Conclusion

This study shows that the combination of low dose of ABZ and 2ME represent a synergistic anti-tumor effect. High effectiveness of the combination therapy stemmed from the inhibition of the proliferation of tumor cells, suppression of angiogenesis, and induction of apoptosis. The finding that the combination of two colchicine-domain binders can act synergistically, suggests that such drug combinations, which would not normally be considered due to the similar mechanism of action and identical binding sites may nevertheless provide therapeutic benefit in cancer therapy.

## Competing interests

The authors declare that they have no competing interests.

## Authors’ contribution

AE designed and carried out the experiments and prepared the manuscript. PG supervised the project and contributed in experimental design and interpreting the data. KP participated in data analysis and revised the manuscript. DLM conceived the study, participated in its design, and edited the manuscript. All authors read and approved the final manuscript.

## Authors’ information

^1^Cancer Research Laboratories, Department of Surgery, University of New South Wales, St. George Hospital, Sydney, NSW, Australia, ^2^Cancer Pharmacology and Therapeutic Laboratory, Medical Oncology, St. George Hospital, Sydney, NSW, Australia

## Pre-publication history

The pre-publication history for this paper can be accessed here:

http://www.biomedcentral.com/1471-2407/13/86/prepub
